# Positive selection on the MHC class II *DLA-DQA1* gene in golden jackals (*Canis aureus*) from their recent expansion range in Europe and its effect on their body mass index

**DOI:** 10.1186/s12862-021-01856-z

**Published:** 2021-06-16

**Authors:** Milomir Stefanović, Duško Ćirović, Neda Bogdanović, Felix Knauer, Miklós Heltai, László Szabó, József Lanszki, Chavdar Dinev Zhelev, Helmut Schaschl, Franz Suchentrunk

**Affiliations:** 1 Department of Biology and Ecology, Faculty of Sciences, University of Novi Sad, Trg Dositeja Obradovića 2, 21000 Novi Sad, Serbia; 2 Faculty of Biology, University of Belgrade, Studentski trg 16, 11000 Belgrade, Serbia; 3 Research Institute of Wildlife Ecology, University of Veterinary Medicine Vienna, Savoyenstrasse 1, 1160 Vienna, Austria; 4 Institute for Wildlife Conservation, Szent István University, Páter Károly utca 1, Gödöllő, 2100 Hungary; 5 Ecological Research Group, University of Kaposvár, PO Box 16, 7401 Kaposvár, Hungary; 6 Southwest State Forestry Enterprise, 2700 Blagoevgrad, Bulgaria; 7 Department of Evolutionary Anthropology, University of Vienna, Althanstrasse 14, 1090 Vienna, Austria

**Keywords:** Golden jackals, MHC, DLA, Serbia, Bulgaria, Hungary, Adaptation, Selection

## Abstract

**Background:**

In Europe, golden jackals (*Canis aureus*) have been expanding their range out of the southern and southeastern Balkans towards central Europe continually since the 1960s. Here, we investigated the level of functional diversity at the MHC class II *DLA-DQA1* exon 2 in golden jackal populations from Bulgaria, Serbia, and Hungary. Specifically, we tested for positive selection on and geographic variation at that locus due to adaptation to supposedly regionally varying pathogenic landscapes. To test for potential fitness effects of different protein variants on individual body condition, we used linear modeling of individual body mass indexes (bmi) and accounted for possible age, sex, geographical, and climatic effects. The latter approach was performed, however, only on Serbian individuals with appropriate data.

**Results:**

Only three different *DLA-DQA1* alleles were detected, all coding for different amino-acid sequences. The neutrality tests revealed no significant but positive values; there was no signal of spatial structuring and no deviation from the Hardy–Weinberg equilibrium across the studied range of expansion. However, we found a signal of trans-species polymorphism and significant test results for positive selection on three codons. Our information-theory based linear modeling results indicated an effect of ambient temperature on the occurrence of individual *DLA-DQA1* genotypes in individuals from across the studied expansion range, independent from geographical position. Our linear modeling results of individual bmi values indicated that yearlings homozygous for DLA-DQA1*03001 reached values typical for adults contrary to yearlings carrying other genotypes (protein combinations). This suggested better growth rates and thus a possible fitness advantage of yearlings homozygous for DLA-DQA1*03001.

**Conclusions:**

Our results indicate a demographic (stochastic) signal of reduced *DLA-DQA1* exon 2 variation, in line with the documented historical demographic bottleneck. At the same time, however, allelic variation was also affected by positive selection and adaptation to varying ambient temperature, supposedly reflecting geographic variation in the pathogenic landscape. Moreover, an allele effect on body mass index values of yearlings suggested differential fitness associated with growth rates. Overall, a combination of a stochastic effect and positive selection has shaped and is still shaping the variation at the studied MHC locus.

**Supplementary Information:**

The online version contains supplementary material available at 10.1186/s12862-021-01856-z.

## Background

The golden jackal (*Canis aureus*) is one of the most widespread canid species inhabiting many areas in southern Asia and relatively recently also in southeastern, central, and eastern Europe [1]. In Europe, golden jackals have historically been restricted to some occurrences on the Balkans where they have experienced a drastic decline until the 1960s due to habitat loss and overhunting. Their subsequent recovery in the 1960s and 1970s under legislative protection has eventually led to an initially slow but continuous northward expansion of their range towards central Europe from the 1980s onward (e.g., [2]) with the latest reproduction record from as far north as Poland [3] and beyond. That expansion has raised concerns on their significant role as potential hosts of several pathogens with diverse transmission routes and as a wildlife reservoir of diseases and changes in the disease epidemiology among other European canids [4]. Specifically, the large variety of parasites of golden jackals is being considered a consequence of their wide geographic range, territorial mobility, and a very unselective diet [5]. Since pathogenic selection may vary over space and time due to various ecological factors, such as community structure, prevalence of intermediate hosts, and habitat-specific transmission rates, the immunogenetic diversity of those expanding golden jackal populations may reflect a response to adaptation to likely varying regional pathogenic landscapes.

Genetic variation at functionally important genes, such as those of the multigene family of the Major Histocompatibility Complex (MHC), is considered important for resistance against pathogens, increasing individual fitness and long-term population survival (e.g., [6]). In canids, the MHC genes are referred to as DLA or “dog leukocyte antigen system”. They code for cell-surface proteins that bind to antigens and present them to T-cells, which initiate a cascade of immune responses. MHC genes exist in two main subfamilies: class I genes associated with responses mainly to viruses and class II genes associated with responses to bacteria and parasites (e.g., [7]). High polymorphism at MHC loci, especially in the peptide binding regions, is maintained by balancing selection, as a response to resistance to diverse arrays of pathogens (e.g., [6, 8]). Variability of class II genes has been characterized for several canid species, such as dogs, *Canis lupus f. familiaris* (e.g., [9, 10]), wolves*, Canis lupus,* (e.g., [11–16]), African wild dogs, *Lycaon pictus*, (e.g., [17, 18]), coyotes, *Canis latrans*, [11, 19], arctic foxes, *Vulpes lagopus*, [20], and red foxes, *Vulpes vulpes*, [21]. However, so far only one study has examined MHC variability in golden jackals from a restricted geographical range in the Balkans [22]. In the latter study, the two examined populations from Croatia were considered to still contain enough functional MHC diversity necessary for adequate immune responses to pathogen peptides, despite allelic diversity at the studied MHC class II genes *DLA-DRB1, DQA,* and *DQB1* was shown to be clearly lower than in grey wolves [22].

Several diseases in canids can be related to specific MHC alleles or to increased MHC homozygosity [23–25]. On the other hand, as MHC genes are important for pathogen resistance and usually show signatures of selection [26–28], certain alleles and/or genotypes may be important for individual fitness and life-time reproductive success of their carriers. Arbanasić et al. [22] have revealed positive selection on all three examined MHC class II genes of the golden jackals from Croatia, namely on two codons within *DLA-DQA1*, eleven codons within *DLA-DRB1* and nine codons within the *DLA-DQB1* gene. Niskanen et al. [15], who based on the analyses of association between helminth prevalence and diversity in MHC genes at the allele and SNP level, showed that wolves carrying specific MHC allele, SNP haplotypes and SNP alleles had fewer helminth infections. On the other hand, climatic differences, such as ambient temperature, humidity, and precipitation, may influence not only the geographic distribution and population density of hosts (i.e., jackals), but also that of their pathogens and the associated vectors [29]. Therefore, local or regional, as well as temporal immunogenetic adaptation of the jackals may be controlled to some extent by geographically varying pathogen distributions, which would be expected to be indirectly shaped by different climate regimes at the micro or macro-geographical scale.

In the present study, we investigated the level of functional diversity at the MHC class II *DLA-DQA1* exon 2 in the golden jackal populations that have over the last 50–60 years expanded continuously from Bulgaria into Serbia, and presumably also from there into Hungary [2, 30]. It appears that currently the whole expansion range across Bulgaria, Serbia, and Hungary is genetically connected by relatively high gene flow (around 1.0 migrants per generation) as indicated by putatively neutrally evolving microsatellite loci [30]. Therefore, we firstly expected a higher diversity of protein variants at the DLA-DQA1 locus than in the jackals from the smaller study area of Croatia published by Arbanasić et al. [22]. Secondly, despite the relatively high gene flow but under positive selection and given the high diversity of pathogens across the golden jackal’s geographic range [5], we also expected some significant geographic variation in the *DLA-DQA1* exon 2 protein frequencies due to adaptation to supposedly regionally varying pathogenic landscapes. We also tested for varying selection regimes along the expansion route from Bulgaria towards Serbia and further on towards Hungary. Particularly for the Hungarian population that has been established very recently, but also to some extent for the Serbian jackals, we could expect a high stochastic colonization effect on the presence of protein variants, hence perhaps no signal of positive selection. In the face of the generally high level of neutral gene flow mentioned above, a significant spatial differentiation at the DLA-DQA1 locus would also correspond to the hypothesis of diversifying selection. Contrary, a more or less homogenous pattern of variation across the expansion area would be in favor of balancing selection or/and a comparatively weak selection effect, relative to the spatially homogenizing effect of high gene flow (compare e.g., [16]). Moreover, specifically climate-related variation of individual combinations of protein variants resulting from homozygous or heterozygous genotypes (as e.g. reported by Awadi et al. [31] for hares from an ecocline) would support the hypothesis of diversifying (positive) selection, as climatic parameters may indicate specific environmental conditions favorable or unfavorable for diverse pathogens or their vectors, creating different selective landscapes for the jackals.

Furthermore, for the golden jackals from Serbia, of which body weight, head body length, age category, and sex data were available, we examined the potential effect of the different DLA-DQA1 protein variants on individual body weights relative to the respective head body lengths by accounting for possible age and sex effects and by statistically controlling for unknown locality-related effects (“black box”), such as varying pathogenic infections or food resources. Specifically, we tested for higher individual body mass indexes (i.e., a combination of body weight and head body length) in jackals with a combination of different DLA-DQA1 protein variants, corresponding to the “overdominance hypothesis”, rather than in jackals with identical protein variants, corresponding to the “allelic advantage hypothesis” (e.g., [8]), against no such effects, matching the null hypothesis. Especially under diversifying selection, we expected a signal in favor of the “overdominance hypothesis”, since jackals carrying heterogeneous protein variants might possibly trigger an immune response to remove a higher diversity of pathogens more effectively and thus might be able to allocate a proportionally higher amount of energy to other than immunological performances, such as growth.

## Results

**Fig. 1:**
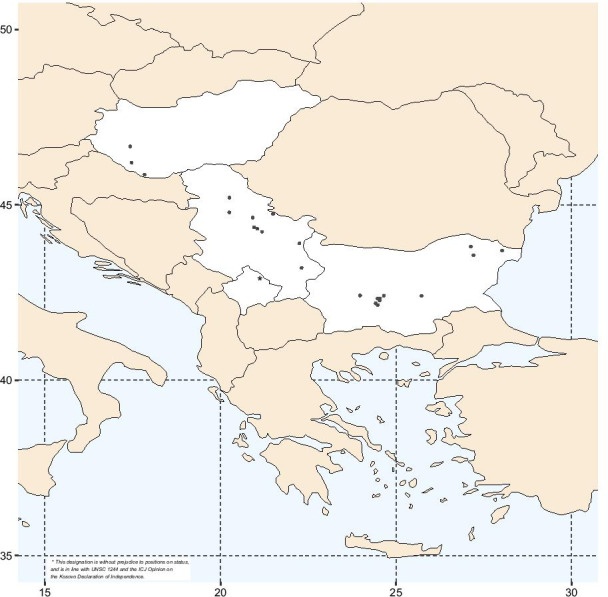
Geographic positions of sampling localities of *Canis aureus* individuals (black dots) from Bulgaria, Serbia, and Hungary, indicating also the historical expansion starting from the southeastern Balkans toward central Europe from the 1970s onward until today. The map was made by the freely available software R [32] using *maps* [33] and *ggplot2* [34] packages

**Fig. 2:**
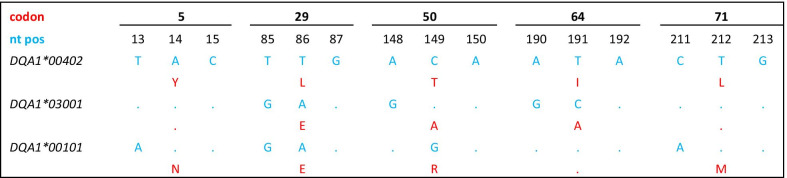
Nucleotide (in blue) and amino-acid (in red) alignments of polymorphic positions in *DLA-DQA1* sequences in golden jackals

**Fig. 3:**
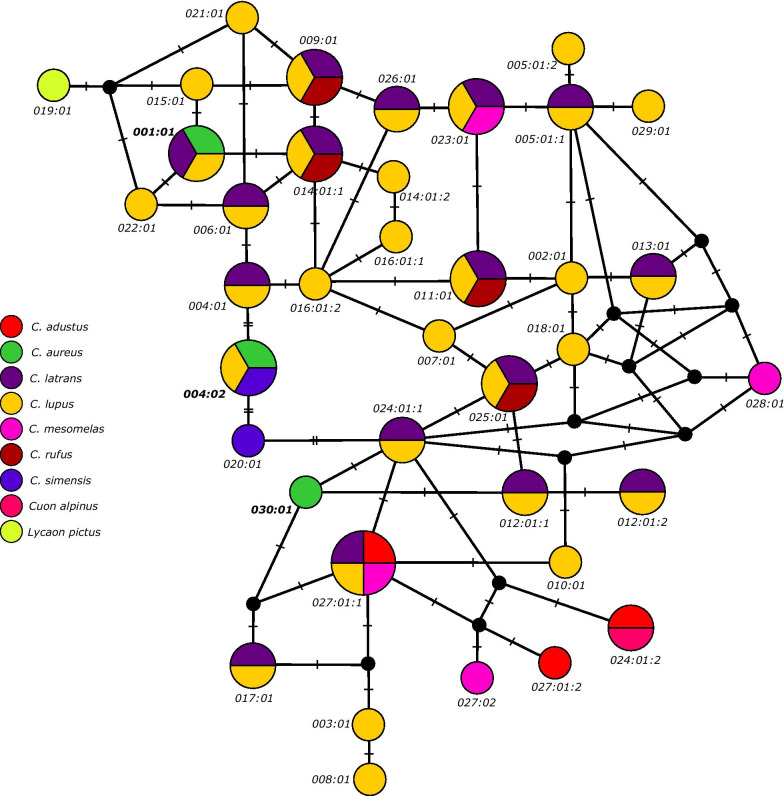
Evolutionary relationships among *DLA-DQA1* alleles in Canids, as represented by a median-joining network. For simplicity, allele designations are shown only by numbers (the correct full labels would include “DLA-DQA1*” prior to the respective allele number). Black circles represent median vectors. Alleles are colored according to their presence in species, and further information is given in Additional File 1: Table S1

In total, of all 164 golden jackals studied from Bulgaria, Serbia and Hungary (Fig. 1), only three different *DLA-DQA1* alleles based on eight polymorphic nucleotide positions were detected (Fig. 2); all of them were already found previously in canid species (Fig. 3). The most prevalent allele was DLA-DQA1*004:02 (hereinafter referred to as DLA-DQA1*00402) with a frequency of 0.707, the second most prevalent allele was DLA-DQA1*030:01 (as DLA-DQA1*03001) with a frequency of 0.287, whereas the allele DLA-DQA1*001:01 (as DLA-DQA1*00101) had a frequency of 0.006 and was presented as a single copy allele in two heterozygous individuals from Serbia. So far, only the DLA-DQA1*03001 allele was exclusively found to be typical for golden jackals [22], while the allele DLA-DQA1*00402 was already shown to be widely distributed in golden jackals from Croatia [22]. However, the latter allele was also identified in various dog breeds [35] and Ethiopian wolves [36]. The allele DLA-DQA1*00101 was also identified in a number of dog breeds [35], in grey wolves from Croatia [14], and for the first time was reported in this study in golden jackals. Fifty percent of all of the examined individuals were homozygous for the DLA-DQA1*00402 allele*,* while 7.32% were homozygous for DLA-DQA1*03001, with the remaining individuals being heterozygous (42.68%). The observed heterozygosity (H_o_ = 0.427) did not differ significantly from the expected heterozygosity (H_e_ = 0.419), with an overall *F*_*IS*_ = -0.019 (95% CI: − 0.161–0.119).

The haplotype diversity of the comprehensive data set amounted to 0.419 ± 0.022, the nucleotide diversity amounted to 0.008, and the average number of pairwise nucleotide differences amounted to 2.098. Based on the Jukes-Cantor evolutionary model, the highest pairwise distance of 2.48% was found between DLA-DQA1*03001 and DLA-DQA1*00101, whereas identical values of 2.06% were found between DLA-DQA1*00402 and DLA-DQA1*03001 as well as DLA-DQA1*00402 and DLA-DQA1*00101. All three detected alleles were coding for different amino-acid sequences (herein after named proteins variants), based on five polymorphic codon positions (Fig. 2). Out of eight segregating sites, all positions were nonsynonymous changes. The highest Poisson pairwise amino-acid sequence distance of 5.01% was found between DLA-DQA1*00402 and DLA-DQA1*00101, as well as between DLA-DQA1*00101 and DLA-DQA1*03001, while a distance of 3.73% was found between DLA-DQA1*00402 and DLA-DQA1*03001.

For the overall dataset, the neutrality tests revealed no significant but positive values for Tajima’s D (1.393), Fu and Li’s D* (1.171) and the F* (1.502) test signaled balancing selection. The same results were obtained when testing for each regional group separately, namely: Bulgaria (Tajima’s D (1.531), Fu and Li’s D* (1.071) and the F* (1.429)), Serbia (Tajima’s D (1.503), Fu and Li’s D* (1.213) and the F* (1.569)), and Hungary (Tajima’s D (1.641), Fu and Li’s D* (1.067) and the F* (1.469)). Furthermore, the overall rate of nonsynonymous substitutions per nonsynonymous site was significantly higher than the rate of synonymous substitutions per synonymous site as revealed by the Z-test (*p* value = 0.0435; Nei-Gojobori method with Jukes Cantor correction); this indicated that the *DLA-DQA1* locus in the golden jackals from the current study region have evolved under positive selection. Significant values for the Z-test were also detected when testing for each regional group (Bulgaria (0.0493), Serbia (0.0434), Hungary (0.0456)). Furthermore, our omegaMap analysis indicated three codons under positive selection, namely in Bulgaria and Hungary codons 29 and 64, and in Serbia codons 29, 50, 64 with a posterior probability for positive selection higher than 95%. No significant signal of recombination was found in the currently studied *DLA-DQA1* golden jackal sequences based on the RDP approaches (RDP, GENECONV, MAXCHI, CHIMAERA and 3SEQ). Furthermore, the *R*_M_ value was estimated to be zero. According to Bondinas et al. [37] all codons under positive selection are located within the antigen binding region, namely codon 29 in the ß strand 3 of peptide pocket 1, codon 50 in the extended chain of pocket 1, and codon 64 in the helix of peptide pocket 6.

Our MJ network clearly indicated trans-specific polymorphism for the *DLA-DQA1* phylogeny (Fig. 3), as shared alleles were observed between different species, and alleles from different species appeared evolutionarily closer than alleles from the same species. According to the current GenBank data (latest access on August 28^th^ 2020) and the available literature, only DLA-DQA1*03001 has been found exclusively in golden jackals so far. All of the alleles were coding for different protein variants, except for the following pairs of alleles: DQA1*005:01:01 and DQA1*005:01:02; DQA1*012:01:01 and DQA1*012:01:02; DQA1*014:01:01 and DQA1*014:01:02; DQA1*016:01:01 and DQA1*016:01:02; DQA1*024:01:01 and DQA1*024:01:02; DQA1*027:01:01 and DQA1*027:01:02. Our MJ network indicated that almost all protein variants differed only by one amino-acid change when there was a direct evolutionary pathway between variants, except between protein variants DQA1*00402 and DQA1*020:01, where two amino-acid changes were observed. Furthermore, in a few cases where evolutionary pathways did connect neighboring variants via median vectors, more than one amino-acid change was observed.

The results of our sPCA indicated neither a significant global (*P* = 0.616) nor any local (*P* = 0.721) geographic structure in the distribution of the genetic variation at the studied locus. Our non-rotated PCA of the temperature data based on the correlation matrix resulted in two principal components that together explained 79.92% of the original variable variance. The first temperature factor (tf1) explained 58.32% and the second temperature factor (tf2) 21.6%. Based on the respective loadings of the original variables, tf1 could be interpreted as a general temperature factor, particularly emphasizing temperature during the warm and cold periods (not the wet or dry seasons), whereas the tf2 reflected the temperature of the wettest period of the year. The non-rotated PCA of the precipitation data extracted one principal component (pf) representing 83.97% of the original variable variance, and could be summarized as a general precipitation factor. Our multinomial modeling of the local climatic differences on the three most common genotypes (Table 1) indicated the highest importance of tf2 in both of the two separate model runs (one accounting for latitude, the other accounting for longitude—see Material and Methods).

**Table 1:** Summary results of the multinomial logistic regression modeling of the occurrence of DQA1 genotypes in the currently studied golden jackals after model averaging

Variable		Latitude		Longitude	
		coefficient	RVI	coefficient	RVI
tf1	2	0.067	0.11	0.054	0.11
	3	0.042		0.046	
tf2	2	− 0.486	**0.90**	− 0.478	**0.88**
	3	− 0.503		− 0.515	
pf	2	− 0.201	0.20	− 0.066	0.19
	3	0.262		0.287	
Lat/Lon	2	0.065	0.12	0.079	0.18
	3	-0.058		-0.015	

The reference category is the homozygous DLA-DQA1*00402 genotype, 2 is heterozygous, and 3 is homozygous for alleles DLA-DQA1*03001. All model averaged coefficients are shown along with the respective relative variable importance (RVI) values. Variables with RVI values above 0.7 are conventionally considered as a key variable of statistically meaningful importance. *tf1* stands for temperature factor 1, *tf2* temperature factor 2, *pf* precipitation factor, *Lat* latitude, *Lon* longitude. Statistically meaningful variables are shown in bold

**Fig. 4:**
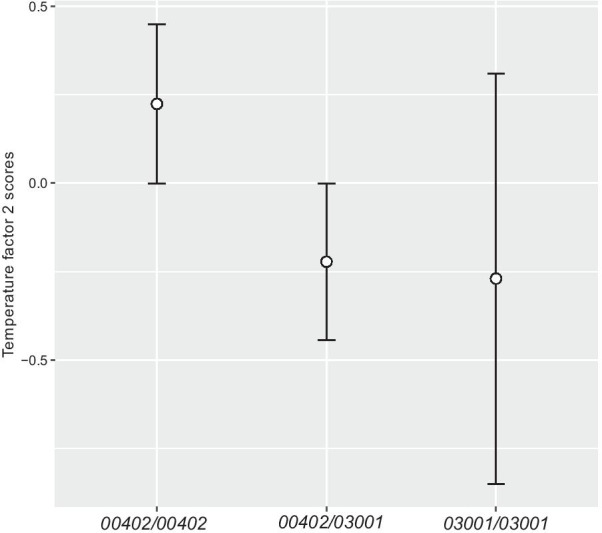
Means of temperature factor 2 scores: means and 95% confidence intervals for three DLA-DQA1 genotypes. Data was plotted using the *ggplot2* [34] package

According to those two model results, there was a clear positive effect of the tf2 on the presence of individual *DLA-DQA1* genotypes in golden jackals from the current study range, independent of the geographical position of the jackals (i.e., independent of potential other unknown locality-related factors). Namely, the heterozygous individuals were more often occurring at locations with lower temperatures during the wettest period of the year (Fig. 4), as their mean was well below the mean of individuals homozygous for allele DLA-DQA1*00402, and that was also statistically confirmed by non-overlapping 95% confidence intervals for those two genotypes. The same result was in principle obtained for the individuals homozygous for DLA-DQA1*03001, even though the respective confidence interval was larger and included the mean of the individuals homozygous for allele DLA-DQA1*00402.

**Fig. 5:**
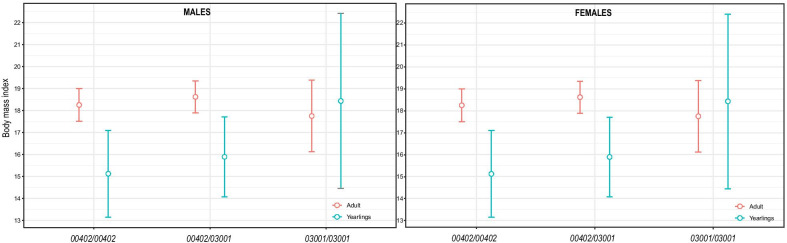
Predicted body mass index (bmi) values (y-axsis) as a function of DLA-DQA1 genotypes, derived from generalized linear modeling with DQA1 genotypes, sex and age class as predictor variables. Error bars are showing 95% confidence intervals. Data was plotted using the *ggplot2* [34] package

The bmi values estimated by model averaging did not differ among the genotypes in adults (Fig. 5), but were significantly lower for yearlings homozygous for DLA-DQA1*00402 and for heterozygous yearlings (no overlap of the 95% CIs). Contrary, the estimated bmi means of yearling males and females homozygous for DLA-DQA1*03001 were above the upper bounds of the 95% c.i. of the other two genotypes and very similar to the values of all genotypes in adults. However, the 95% c.i. ranges of DLA-DQA1*03001 homozygote yearlings were very large and overlapping with those of the other two yearling genotypes, most likely due to the very small sample size for DLA-DQA1*03001 homozygote yearlings. Thus, yearlings homozygous for DLA-DQA1*03001 most likely reached bmi values typical for adults already as yearlings, which could indicate a faster growth rate. According to our model averaging, except for age class and genotype, all other explanatory variables in our global model did not have a statistically meaningful effect on the bmi values (see Table 2).

**Table 2:** Relative variable importance (RVI) values of predictor variables used in the body mass index (bmi) generalized linear models after model averaging. Statistically meaningful predictor variables for bmi are shown in bold

	Age	dqagt	Age * dqagt	Lat	Lon	Lat*Lon	Sex	tf1	pf	tf2
RVI	**1**	**0.74**	0.68	0.62	0.55	0.40	0.36	0.30	0.26	0.24

*dqagt* DQA genotype, *Lat* latitude, *Lon* longitude, *tf1* temperature factor 1, *tf2* temperature factor 2, *pf* precipitation factor

## Discussion

The currently observed genetic diversity at the DLA-DQA1 locus of golden jackals from the Balkans and Hungary is obviously shaped by three mutually not exclusive processes: (a) a long term operating selection in the past as evident from the trans-species polymorphism signals and positive selection signals as obtained from site-specific test results, independent from recombination; (b) a stochastic effect due to a (supposedly severe) bottleneck period prior to the recent expansion phase; and; (c) molecular adaptation to ambient temperature as well as a putative fitness advantage reflected by an allele effect on the body mass index of growing jackals. Overall, we found a low level of *DLA-DQA1* exon 2 sequence variability. This is in accordance with previous population genetic studies in golden jackals from Europe that indicated very low genetic diversity and shallow spatial structuring based on mitochondrial DNA sequences and multilocus microsatellite data [38–41]. Moreover, previous studies in MHC genes of canids indicated generally the highest diversity for the DLA-DRB locus, whereas the lowest diversity was observed in the DQA locus. Nevertheless, clearly higher diversity values than presently found were observed in all three studied DLA class II loci (DRB, DQA, DQB) of dogs and wolves [9, 10, 12–14]. But the previous studies in golden jackals from Croatia [22] and Ethiopian wolves [36] revealed similarly low diversity indices as in our study. Moreover, the study of Galov et al. [42] on hybridization between golden jackals and dogs (*Canis lupus f. familiaris*) revealed only those two DQA alleles in wild living purebred golden jackals that were found to be the two most prevalent ones (i.e., DLA-DQA1*00402, DLA-DQA1*03001) in our present study. Remarkably, the most frequent haplotype/allele DLA-DQA1*00402 in our study had a frequency of 0.707, similar to frequencies found in the golden jackals from Croatia (0.852) or the Ethiopian wolves (0.83). Those high frequencies may indicate already its long-term and large-scale preferences across many diverse habitats and pathogenic landscapes.

Generally, the low genetic diversity observed currently in the golden jackals from southeastern Europe is concordant with their historical population contraction, followed by a recent rapid demographic expansion towards central Europe (e.g. [38, 39]). Arbanasić et al. [22] interpreted the low genetic diversity at the *DLA-DQA1* locus in two likely separated populations in Croatia as having resulted from drift processes rather than a lack of historical positive selection in the golden jackals from Europe.

### Mechanisms to maintain MHC diversity

Even though genetic variability at MHC loci seems to be driven primarily by spatially and timely varying selection pressures from pathogens and parasites, several mechanisms, such as mutations, recombination, genetic drift, gene flow and gene conversion can also affect the diversity at these loci. Neutral genetic diversity is primarily shaped by genetic drift, under the absence of significant gene flow, but how drift affects adaptive variation is not well understood. Some studies indicated a strong effect of drift on MHC functional diversity (e.g. [43, 44]), whereas others failed to prove any drift effect in spite of a strong bottleneck signal [45]. The apparent reduction of neutral molecular diversity as revealed by neutral molecular markers suggests a predominant effect of demographic processes, with drift as an important force in shaping the *DLA-DQA1* diversity of the golden jackals from the Balkans. Having particularly in mind the demographic reduction that they have experienced at least during the twentieth century (see e.g., the overview in [46]), a drift effect on the DLA-DQA1 locus might have happened only very recently in evolutionary terms. However, it is unknown when or where from the golden jackals have colonized the Balkans and how many generations in the past. Zachos et al. [38] have interpreted the low genetic diversity in their studied Serbian populations, as indicated by low heterozygosity and allelic diversity at nine microsatellite markers, as a possible drift effect (founder effect) along the recent expansion by only few individuals from Bulgaria towards Serbia. However, further data based on the same nine markers indicated the same low allelic diversity in the studied Bulgarian populations that had been established already very early in the course of their northwestward expansion [30]. That latter study also included jackals from southeastern Bulgaria (Strandja region) which had served as primary source population of the currently studied population expansion. This latter finding may suggest that the low allelic diversity at this locus has resulted from a clearly earlier genetic bottleneck than the very recent one in Bulgaria during the twentieth century. And this interpretation is supported by the fact that the golden jackals from two Croatian populations supposedly with a demographic history independent from that of the presently studied Bulgarian and Serbian jackals, harbored the same two most prevalent *DLA-DQA*1 alleles (DLA-DQA1*00402, DLA-DQA1*03001). Rather, the demographic bottleneck may have been connected to the colonization of the Balkans by golden jackals, probably not too long ago in historic or prehistoric times. Detailed comprehensive population genetic information on the colonization of the Balkans by golden jackals is not available to date. However, recent morphometric skull analyses [47] suggested the Anatolian Peninsula as a likely source region for all Balkan golden jackals. This assumption is also very plausible in biogeographical terms as the late glacial land bridge between NW Anatolia and the southeastern Balkans that has provided opportunity for gene flow in terrestrial mammals has disintegrated only some 8500 BP; and available mitochondrial DNA data are not incongruent with the hypothesis that golden jackals from the Balkans are originating from the Anatolian Peninsula [48]).

Given the currently revealed signal of three codons under positive selection, but also given the evident pattern of trans-species polymorphism through retention of the same alleles across speciation events, it seems that balancing selection was important for the long-term historical maintenance of diversity at the *DLA-DQA1* locus in golden jackals. This is also supported by the absence of spatial structuring in the DLA-DQA1 locus across the study region, and no deviation from Hardy–Weinberg equilibrium.

### Molecular adaptation to the climate and possible fitness advantages

Golden jackals seem to be very adaptable and plastic in their behavior (e.g., [49]), which may facilitate their expansion in the Balkans towards central Europe [50] and beyond, and their opportunistic dietary habits enable them to use anthropogenic food resources [51]. Our results indicated that individuals heterozygous or homozygous for the allele DLA-DQA1*03001 were more often present in areas with lower ambient temperature during the wettest period of the year than individuals not carrying that allele. Actually, host-parasite dynamics may be affected by climatic and seasonal changes, such as higher temperature may increase both parasite development and infectivity [52]. In this context the straightforward interpretation of the overdominance hypothesis would predict more heterozygous individuals to occur in regions of higher ambient temperature with supposedly higher diversity of the pathogenic landscapes. However, our linear modeling results of the genotype frequencies rather indicated better adaptation to an increased temperature during the wettest period of the year in jackals homozygous for the DLA-DQA1*00402 allele; and this corresponded to an allele rather than an overdominance effect of selection.

Gherman and Mihalca [5] already demonstrated that prevalences of some golden jackal parasites vary across Serbia, Bulgaria, and Hungary. This accords to the assumption of a spatiotemporal heterogeneity of the pathogenic landscape of the golden jackals along their expansion route from the southeastern Balkans toward central Europe, even though this heterogeneity could be blurred to some extent by different sampling seasons [53]. In general, effects of ambient temperature on pathogen diversity and virulence has been demonstrated already in earlier studies on other species, and some of them have revealed increased allelic diversity at MHC loci with higher temperature (e.g. Atlantic salmon, [54]). Also, climate-driven positive selection on MHC genes across relatively short geographical distances along an ecological cline has been shown in cape hares, *Lepus capensis*, from North Africa by Awadi et al. [31]. Therefore, the presently observed effect of ambient temperature on the distribution of homozygous individuals may be interpreted as a signal of regional adaptation to supposedly geographically varying pathogen and parasite selection pressures, particularly in combination with humidity, whereas in environments of high ambient temperature but little humidity pathogen frequencies may even drop, as in extremely arid habitats like in deserts. But as long as no specific details on composite infections of the studied jackal populations and the ecological requirements of infections with those parasites and pathogens, as well as possible selective interactions with other MHC loci are available, no further interpretations can be given.

Thus, despite the very likely reduction of allelic diversity at the DLA-DQA1 locus in the jackals from the Balkans as a consequence of their younger demographic history, our findings strongly suggest an important role of positive selection on the distribution of the remaining protein variants. Remarkably, this positive selection signal is maintained along the whole expansion range from Bulgaria to the recently occupied area in Hungary. Probably, the gene flow across the whole expansion range is sufficient enough [30] to prevent regional isolation and stochastic effects that otherwise could override positive selection signals. In addition to the ambient temperature-related occurrence of genotypes positive selection was also clearly indicated by significant site-specific signals of positive selection as well as the significant effect of genotypes on the individual body mass index of the Serbian jackals. Following Wakeland [55] the ability of a population to respond to pathogens would not necessarily be threatened by reduced MHC variability, if the alleles were still functionally divergent enough. This argumentation corresponds to the “divergent alleles advantage hypothesis” expecting that low locus-specific allelic diversity may still be compensated by an increased number of overall haplotypes as based on several MHC-loci. In principle, such a pattern was observed in the jackals from Croatia, were specific three-locus (DQA, DQB, DRB) haplotypes as well as one DRB1 and one DQB1 allele were found only in the population from Dalmatia despite similar DQA1 frequencies in the two studied populations [22]. Corresponding to the divergent alleles advantage hypothesis, the currently observed three alleles (protein variants) have diverged evolutionarily up to four amino acids from each other, in spite of a very low overall divergence at the amino-acid level in canids according to our MJ network.

Apart from their importance for pathogen resistance, MHC genes are known for their potential influence of maternal–fetal interactions, kin recognition, mate choice, reproductive success, but also to affect the individual fitness and population viability [6, 8]. Several studies in canids indicated an association of particular MHC alleles and/or haplotypes with a higher relative risk of immune-mediated diseases (e.g., [56–58].). Contrary, a heterozygote advantage is generally recognized as a mechanism explaining the diversity in MHC genes, with individuals carrying a higher diversity of alleles being able to recognize a broader range of antigens (“overdominance hypothesis”). However, our results were not in accordance with the overdominance hypothesis; rather, the significant effect of a particular DLA-DQA1 allele (homozygous genotype) on the body mass index (bmi) of yearling jackals were not in disagreement with the “allelic advantage hypothesis”. Even though less pronounced than in red foxes, *Vulpes vulpes*, or wolves, *Canis lupus*, a sexual size dimorphism is noticeable in golden jackals as well, as already proved for central Bulgarian jackals, with larger bodies and a higher body weight index in males than in females [59]. Presently, we found no significant difference in bmi values between the DLA-DQA1 genotypes among adults of either sex. But both sexes of yearlings homozygous for the allele DLA-DQA1*03001 had higher bmi values than yearlings carrying other genotypes, and that indicating a faster growth rate for the DLA-DQA1*03001 homozygotes*.* This may suggest an advantage of those individuals during their growth period, possibly due to better fighting certain pathogens or parasites in their early ontogeny, and thus providing relatively more energy for growth. Obviously, this advantage appeared to be independent of geographic location and local climate (i.e., possibly varying local pathogenic scenarios). On the other hand, MHC heterozygous mammals may tend to be co-infected by fewer parasites than homozygotes, as demonstrated in a study of MHC heterozygote superiority in water voles, *Arvicola terrestris*, against multiple parasites in natural populations [60]. In general, MHC heterozygosity seems to increase fitness within wild populations, as some studies indicated that MHC heterozygosity was positively correlated with body mass and resistance to pathogens, irrespective of genome wide-heterozygosity (e.g. in Alpine ibex, [61] and Alpine chamois [62]). However, as to our knowledge no meta-analysis is available on that topic. At least our present results are rather in opposition to this hypothesis, but no individual infection data were available, and we leave it still open to what extent our conclusion would be supported by other MHC loci.

## Conclusions

The presently studied golden jackals from large parts of their recent expansion range on the Balkans as well as from the by now well established reproducing population of Hungary exhibit on the one hand a demographic signal of reduced *DLA-DQA1* exon 2 variation, specifically reflecting a historical demographic bottleneck. On the other hand, variation at this locus is also affected by positive selection and adaptation to varying ambient temperature, possibly reflecting climate-related geographic variation in the pathogenic landscape. Moreover, our statistical modeling of the occurrence of protein variants or a combination of them at locations with different ambient temperature suggested an allele effect rather than an overdominance effect in terms of selection according to ambient temperature. An allele effect was also found for body mass index values in yearlings, independent of climate parameters or geographical location, suggesting a better growth rate in individuals homozygous for the DLA-DQA1*03001 allele.

## Methods

### Sample collection and laboratory analyses

A total of 164 golden jackal samples (Fig. 1) from several locations across Bulgaria (34), Serbia (95) and Hungary (35) were obtained by regular hunting management activities. Total genomic DNA was extracted using the GenElute Mammalian Genomic DNA Miniprep kit (Sigma-Aldrich, USA) following the manufacturer protocol. The full length exon 2 of the *DLA-DQA1* gene was amplified using the primers DQAin1 and DQAin2 [63], which span intron 1 and intron 2. PCR reactions were carried out in 25 µL volumes and contained 1X Phusion HF buffer, 0.2 µM of each primer and 0.2 units of Phusion High-Fidelity DNA Polymerase (Thermo Fischer Scientific, USA). The thermal profile consisted of 5 min at 98 °C, followed by 30 cycles at 98 °C for 10 s, 58 °C for 20 s and 72 °C for 15 s, with a final 8 min elongation at 72 °C. PCR products were purified using carboxyl-modified magnetic beads. Sanger sequencing was performed bi-directionally using the same primers as described above on an automated ABI 3130 DNA Analyzer (Applied Biosystems, USA). The obtained sequences were edited in the SeqScape software (Applied Biosystems, USA), where each polymorphic position was examined by eye and coded as corresponding IUPAC code for ambiguous sites, while allele determination was done by haplotype reconstruction in PHASE [64] as implemented in DnaSP v6 [65], based on five replicate runs each of 1000 iterations with 1000 burn-in steps. To improve the phasing results, a reference library of previously published *DLA-DQA1* jackal alleles was used along with the homozygous sequences obtained in this study. All alleles obtained after phasing were designated following the rules set out by the DLA Nomenclature Committee [66].

### Molecular diversity parameters estimation

Allele frequencies as well as observed and expected heterozygosity were calculated in Genetix [67], while GenePOP [68] was used to test for deviation from Hardy–Weinberg equilibrium and presence of linkage disequilibrium. Molecular diversity indices based on sequence data were calculated in DnaSPv6, which has also been used to calculate Tajima’s D to test for departure from neutral expectations. MEGA6 [69] was used to calculate the overall pairwise nucleotide distances and average pairwise amino acid distances corrected for the suggested nucleotide evolutionary model, based on 1000 bootstrap replicates.

### Selection and recombination analyses

MEGA was also used to test for selection based on the calculation of the nonsynonymous (dN) and synonymous (dS) substitution rates per site based on the Nei-Gojobori method [70] with Jukes-Cantor correction. Significance was examined by a codon-based Z test after 1000 bootstrap replications. Furthermore, in order to account for possible effects of recombination, positive selection was also tested with the software omegaMap [71]. Two Markov chain Monte Carlo tests were run for 500,000 iterations, with a 50,000 iterations burn-in. The probable values of the mutation rate (μ) and the transversion/transition rate ratio (k) were adjusted to follow an improper inverse distribution, with starting values for μ and k set at 0.1 and 3, and the selection parameter (ω) and the recombination rate (ρ) adjusted to follow inverse distributions in the range between 0.01 and 20 for ω and 0.01 and 100 for ρ. The value of ω was estimated for each codon independently and ρ as a block-like structure of 10 codons. Furthermore, the presence of recombination and locations of recombination breakpoints were assessed by the RDP package [72], using the RDP, GENECONV, MAXCHI, CHIMAERA and 3SEQ with default settings. The program DnaSP was used to determine the minimum number of inferred recombination events (*R*_M_), which is a minimal number of different positions at which recombination has occurred.

### Spatial analyses

In order to assess the spatial distribution of *DLA-DQA1* allele variability, a spatial principal component analysis (sPCA) was performed as implemented in the R [32] package *adegenet* [73]. The sPCA analysis could indicate the existence of a global structure (resulting from positive autocorrelation) and/or local structure (resulting from negative autocorrelation), the significance of which was tested by a Monte-Carlo permutation test. In order to infer the evolutionary relationships between the presently recovered *DLA-DQA1* alleles, we constructed a median-joining (MJ) network using the PopArt software [74], by including all the Canids alleles available in the online database IPD-MHC Database (https://www.ebi.ac.uk/ipd/mhc/, accessed latest on August 28th 2020; Additional file 1: Table S1).

### Climatic and bmi statistical modeling

In order to test for potential effects of regional climatic differences on the spatial distribution of individual DLA-DQA1 genotypes the statistical software R was used to run multinomial logistic models (for the two most frequent alleles, see “Results” section). The respective genotype category (homozygous for the first allele, homozygous for the second allele, heterozygous genotype) was used as dependent variable, whereas climatic data (see below) and geographic coordinates (longitude, latitude) were used as explanatory variables. Latitude and longitude, however, were highly positively correlated (Pearson´s *r* = 0.919) for our sampling localities, which prevented including them as interaction factor in the models, as that would have created a serious multicollinearity problem. Nevertheless, no serious correlations (*r* < 0.05) were found for longitude and the climate variables as well as latitude and the climate variables, which allowed for running two separate sets of models, one with the climate variables and latitude in the global model and one with longitude and the climate variables. For the climatic data, location-specific values were obtained from the WorldClim—Global Climate Data database [75] at the resolution of 30 s using the R package *raster* [76]*,* based on the individual latitude and longitude information, which were jittered to provide random noise to geographic data. All downloaded bioclimatic variables were *ln*-transformed to reduce their variances, and two principal components analyses (PCA) were performed, the first one reducing the temperature related bioclimatic variables (BIO1, 5, 6, 8, 9, 10, 11) and, the second one reducing the precipitation related bioclimatic variables (BIO12-19, except BIO 15). Both PCAs were based on unrotated solutions and on the correlation matrix. The syntax of the first global multinomial model was: *genotype* ~ *temperature factor 1* + *temperature factor 2* + *precipitation factor* + *latitude* and that of the second was: *genotype* ~ *temperature factor 1* + *temperature factor 2* + *precipitation factor* + *longitude.* The MuMIn R library [77] was used to perform model selection and model ranking according to AIC values corrected for small sample size with the “*dredge*” function, while the “*model.avg”* function was used for model averaging (including the model averaged estimates) for each parameter, together with the respective relative variable importance (RVI). Only variables with RVI values higher than 0.7 were considered as statistically meaningful and therefore used in our model interpretation.

For Serbian jackals, of which additional information on body weight (bw), head body length (hbl), age class and sex was available, generalized linear models (glm) were run on the individual scores of the body mass index (bmi) of the jackals to test for a possible effect of DQA1 genotypes (three categories for the two most prevalent protein variants—see “Results“ section), together with longitude, latitude, age class, sex, and climatic variables as predictors, on the bmi as response variable. In particular, we used the following global syntax: *glm (bmi* ~ longitude * latitude + dqa genotype * age category + dqa genotype * sex class + *temperature factor 1* + *temperature factor 2* + *precipitation factor)*. The individual bmi was calculated in parallel to the human bmi as bw [kilogram]/hbl^2^ [meter]. The individual DLA-DQA1 genotype had three categories: homozygous for allele *DLA*-DQA1*03001, homozygous for allele DLA-DQA1*00402, and heterozygous for both alleles. The age categories used were “adults” and “yearlings” following Ćirović et al. [78]. Longitude and latitude could be used simultaneously for the modeling, as for the Serbian data set there was no significant correlation, which would have otherwise indicated a multicollinearity problem. We used the *dredge* and *model.avg* functions for RVI values. The interpretation of our model results was based on the RVI values of the determining factors and variables, and the estimated averages of their coefficients and respective 95% c.i. values.

## Supplementary Information

**Additional file 1. Table S1.** List of *DLA-DQA1 *Canid alleles downloaded from the IPD-MHC database.

## Data Availability

The genetic data produced in the current study are already freely available from previous publications via GenBank (https://www.ncbi.nlm.nih.gov/genbank/), and can be accessed under the following accession numbers: AJ311099, KT159767, U44786. Furthermore, data can be accessed at the IPD-MHC database, available at https://www.ebi.ac.uk/ipd/mhc/, under the following accession numbers: DLA08117, DLA08122, and DLA08223.

## Abbreviations

Bmi: Body mass index
BP: Years before present
bw: Body weight
DLA: Dog leukocyte antigen system
glm: Generalized linear models
hbl: Head body length
MHC: Major Histocompatibility Complex
MJ: Median-joining network
PCA: Principal components analyses
RVI: Relative variable importance
sPCA: Spatial principal component analysis
