# Supplementary material for: Positive selection on the MHC class II DLA-DQA1 gene in golden jackals (Canis aureus) from their recent expansion range in Europe and its effect on their body mass index
Source: BMC Ecol Evol. 2021 Jun 16;21:122. doi: 10.1186/s12862-021-01856-z (PMC8207625; doi:10.1186/s12862-021-01856-z)
Supplement: Supplementary file 1 — Additional file 1. Table S1. List of DLA-DQA1 Canid alleles downloaded from the IPD-MHC database. [file 12862_2021_1856_MOESM1_ESM.pdf]

Table S1 List of *DLA-DQA1* Canid alleles downloaded from the IPD-MHC database available at https://www.ebi.ac.uk/ipd/mhc/, as on status last accessed on August 28th 2020

IPD Accession number	Allele designation	Species
DLA04814	*DLA-DQA1*012:01:2*	*Canis lupus*
DLA04815	*DLA-DQA1*014:01:2*	*Canis lupus*
DLA04816	*DLA-DQA1*016:01:2*	*Canis lupus*
DLA04817	*DLA-DQA1*017:01*	*Canis latrans*
DLA04818	*DLA-DQA1*018:01*	*Canis lupus*
DLA04820	*DLA-DQA1*020:01*	*Canis simensis*
DLA04821	*DLA-DQA1*021:01*	*Canis lupus*
DLA04822	*DLA-DQA1*023:01*	*Canis lupus*
DLA04823	*DLA-DQA1*025:01*	*Canis rufus*
DLA04824	*DLA-DQA1*026:01*	*Canis lupus*
DLA07968	*DLA-DQA1*003:01*	*Canis lupus*
DLA07969	*DLA-DQA1*004:01*	*Canis lupus*
DLA07970	*DLA-DQA1*005:01:1*	*Canis lupus*
DLA07971	*DLA-DQA1*005:01:2*	*Canis lupus*
DLA07972	*DLA-DQA1*006:01*	*Canis lupus*
DLA07973	*DLA-DQA1*007:01*	*Canis lupus*
DLA07974	*DLA-DQA1*008:01*	*Canis lupus*
DLA07976	*DLA-DQA1*009:01*	*Canis lupus*
DLA07977	*DLA-DQA1*010:01*	*Canis lupus*
DLA07978	*DLA-DQA1*011:01*	*Canis lupus*
DLA07979	*DLA-DQA1*012:01:1*	*Canis lupus*
DLA07980	*DLA-DQA1*013:01*	*Canis lupus*
DLA07981	*DLA-DQA1*014:01:1*	*Canis lupus*

DLA07982	*DLA-DQA1*015:01*	*Canis lupus*
DLA08117	*DLA-DQA1*001:01*	*Canis lupus*
DLA08118	*DLA-DQA1*002:01*	*Canis lupus*
DLA08119	*DLA-DQA1*025:01*	*Canis latrans*
DLA08122	*DLA-DQA1*004:02*	*Canis lupus*
DLA08138	*DLA-DQA1*027:01:2*	*Canis adustus*
DLA08141	*DLA-DQA1*027:01:1*	*Canis latrans*
DLA08144	*DLA-DQA1*027:01:1*	*Canis lupus*
DLA08146	*DLA-DQA1*027:02*	*Canis mesomelas*
DLA08149	*DLA-DQA1*022:01*	*Canis lupus*
DLA08150	*DLA-DQA1*029:01*	*Canis lupus*
DLA08151	*DLA-DQA1*023:01*	*Canis mesomelas*
DLA08157	*DLA-DQA1*024:01:2*	*Canis adustus*
DLA08161	*DLA-DQA1*023:01*	*Canis latrans*
DLA08162	*DLA-DQA1*027:01:1*	*Canis mesomelas*
DLA08171	*DLA-DQA1*028:01*	*Canis mesomelas*
DLA08193	*DLA-DQA1*017:01*	*Canis lupus*
DLA08207	*DLA-DQA1*025:01*	*Canis lupus*
DLA08223	*DLA-DQA1*030:01*	*Canis aureus*
DLA08228	*DLA-DQA1*019:01*	*Lycaon pictus*
DLA08236	*DLA-DQA1*027:01:1*	*Canis adustus*
DLA08257	*DLA-DQA1*026:01*	*Canis latrans*
DLA08273	*DLA-DQA1*024:01:1*	*Canis latrans*
DLA08280	*DLA-DQA1*016:01:1*	*Canis lupus*
DLA08281	*DLA-DQA1*024:01:2*	*Cuon alpinus*
